# Increase in invasive *Streptococcus pyogenes* M1 infections with close evolutionary genetic relationship, Iceland and Scotland, 2022 to 2023

**DOI:** 10.2807/1560-7917.ES.2024.29.13.2400129

**Published:** 2024-03-28

**Authors:** Stephen B Beres, Randall J Olsen, S Wesley Long, Ross Langley, Thomas Williams, Helga Erlendsdottir, Andrew Smith, Karl G Kristinsson, James M Musser

**Affiliations:** 1 Laboratory of Molecular and Translational Human Infectious Disease Research, Center for Infectious Diseases, Department of Pathology and Genomic Medicine, Houston Methodist Research Institute and Houston Methodist Hospital, Houston, United States; 2 Departments of Pathology and Laboratory Medicine and Microbiology and Immunology, Weill Cornell Medical College, New York, United States; 3 Department of Paediatric Respiratory and Sleep Medicine, Royal Hospital for Children, Glasgow, Scotland; 4 Department of Child Life and Health, University of Edinburgh, Edinburgh, Scotland; 5 Department of Clinical Microbiology, Landspitali – the National University Hospital of Iceland, Reykjavik, Iceland; 6 Faculty of Medicine, School of Health Sciences, University of Iceland, Reykjavik, Iceland; 7 College of Medical, Veterinary and Life Sciences, Glasgow Dental Hospital and School, University of Glasgow, Glasgow, Scotland; 8 Scottish Microbiology Reference Laboratory, New Lister Building, Glasgow, Scotland

**Keywords:** Molecular Epidemiology, Population Genomics, Infectious Disease, Phylogenetics, Streptococcus

## Abstract

Group A *Streptococcus* isolates of the recently described M1_UK_ clade have emerged to cause human infections in several European countries and elsewhere. Full-genome sequence analysis of M1 isolates discovered a close genomic relationship between some isolates from Scotland and the majority of isolates from Iceland causing serious infections in 2022 and 2023. Phylogenetic analysis strongly suggests that an isolate from or related to Scotland was the precursor to an M1_UK_ variant responsible for almost all recent M1 infections in Iceland.

A new emergent genetic variant of type *emm1* group A *Streptococcus* (GAS) known as M1_UK_ was recently discovered during a period of increased scarlet fever activity and invasive infection notifications in England [1]. The M1_UK_ variant over-expresses streptococcal pyrogenic exotoxin A (SpeA, also known as scarlet fever toxin), a GAS virulence factor [1-3]. The M1_UK_ variant has been identified in several European and North American countries, commonly in conjunction with surges in GAS infections occurring in 2022 and 2023 [4-14]. While investigating recent surges in severe invasive GAS (iGAS) infections in Scotland and Iceland using whole genome sequencing (WGS), we discovered a very close genetic relationship between several M1_UK_ from Scotland and all M1_UK_ isolates from Iceland. Given the geographical separation, this finding was unexpected. The GAS population genomic data are consistent with transmission of a single M1_UK_ strain from Scotland to Iceland, where it spread extensively, causing nearly all M1 invasive infections in 2022 and 2023.

## iGAS infection surge

**Figure 1:**
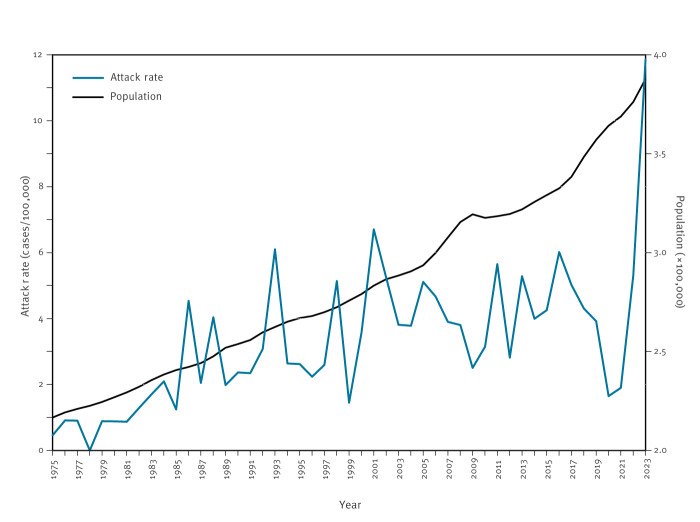
Group A streptococcus invasive infections per 100,000 population in Iceland, January 1975–June 2023 (n = 495)

In Iceland where current iGAS surveillance encompasses the entire populace, iGAS infections increased strongly in the latter half of 2022, extending into the first half of 2023 (Figure 1). The population-adjusted iGAS attack rate for the first half of 2023 (11.9/100,000) was 2.7-fold higher than the mean (4.4/100,000) for the 20 years preceding the COVID-19 pandemic (2000–2019). The increase in iGAS infections was greatest for children younger than 6 years, in whom it increased 8.2-fold from a mean of 1.7 cases per year for 2000–2019 to 14 cases in the first half of 2023. Although the surge involved multiple *emm* types, M1 was most prevalent, causing 48 of 75 iGAS infections in 2023 [10]. Although less comprehensive surveillance information is available for Scotland, a similar substantial increase in iGAS infections in 2022 and 2023 occurred in central Scotland, with M1 isolates being most prevalent and young children disproportionately affected [6,15].

## Whole genome sequencing

**Figure 2:**
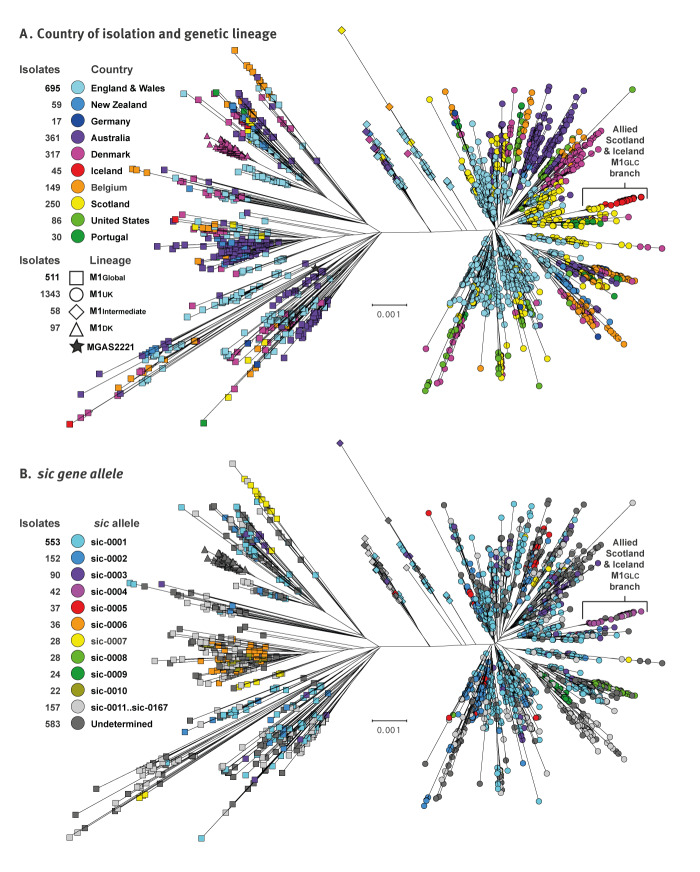
Phylogenetic relationships among contemporary group A streptococcus M1 isolates, 2005–2023 (n = 2,009)

The GAS M1 isolates from Scotland (n = 250; collected 2014–2023) and Iceland (n = 45, collected between November 2022 and November 2023) were cultured from normally sterile sources such as blood or deep tissue, and identified by standard microbiological laboratory procedures at Landspitali (the National University Hospital of Iceland) in Reykjavik or at the Scottish Microbiology Reference Laboratory in Glasgow, where isolates are referred as part of the National invasive GAS surveillance programme. Most Icelandic patients were from the Reykjavik region. Two isolates were cultured from patients hospitalised in Akureyri, the largest community in northern Iceland. The genomes of all isolates from Iceland and Scotland were characterised by Illumina short-read sequencing [16]. In addition, all 45 Icelandic and 10 of the Scottish isolates were characterised using Oxford Nanopore Technology long-read sequencing [16]. To place the strains from Scotland and Iceland within a larger phylogenetic context, we compared them with publicly available WGS data for 1,714 M1 isolates collected in nine countries on three continents between 2005 and 2023 (19 years) (Table). Individual isolate characteristics are listed in Supplementary Table S1. Most of these isolates (n = 1,958, 97%) were collected after the 2010 emergence of the earliest known M1_UK_ isolate. The phylogenetic tree based on the WGS data (Figure 2A) shows two major branches with an M1_Global_ (n = 511) and an M1_UK_ clade (n = 1,343) separated by a subset of genetically intermediate isolates (n = 58 M1_Int_). Of the isolates from Iceland, 41 belonged to the M1_UK_ clade, and all 41 were closely related to one another and nine phylogenetically allied isolates from Scotland. These 50 allied isolates were genetically distinct and located on a separate branch from all other M1_UK_ isolates. The isolates from Scotland were proximal to the isolates from Iceland, showing the Scottish isolates to be the evolutionary precursors.

**Table:** Group A streptococcus epidemiological surveillance cohorts, 2005–2023 (n = 2,009)

Country	M1 isolates	Collection period	Bioproject(s)
England & Wales [1]	695	2009–2016	PRJEB12015 and PRJEB17673
Australia [2]	319	2005–2020	PRJNA872282
Belgium [13]	149	Jan 2020–May 2023	PRJNA10033449
Denmark [10]	317	Jan 2018–Feb 2023	PRJEB62579 and PRJEB62635
Germany [14]	17	Oct 2022–Apr 2023	PRJEB64404
New Zealand [11]	59	2018–2019	PRJNA985396
Portugal [9]	30	Sep 2022–May 2023	PRJEB65018
United States [12]	86	2019–2021	PRJNA395240
Australia [5]	42	2007–2021	PRJNA996294
Scotland (this study)	250	2014–2023	PRJNA1076228
Iceland (this study)	45	2022–2023	PRJNA1076228

Phylogeny for 2,009 GAS M1 isolates by inferred neighbour-joining based on 8,599 core chromosomal SNPs identified by Illumina short-read whole genome sequencing read mapping relative to reference strain MGAS2221 (NCBI accession number CP043530). Panel A: isolates are coloured by country of isolation and shaped by M1 genetic lineage. Panel B: isolates are coloured by the 10 most prevalent *sic* gene alleles in descending order.

With current short read sequencing technology it is problematic to assemble reads containing multiple different repeat domains, such as the *sic* gene, and accurately map them to a reference strain containing such repeat structures. The variants labeled ‘undetermined’ are isolates in which a consensus assembly of the *sic* gene was not obtained using the Illumina short-read-length sequencing data. This is likely because the *sic* gene in these isolates had more repeats or a more complex set of repeats that confounded the SPAdes assembler.

## SIC genetic diversity

The *sic* gene encodes the extracellular streptococcal inhibitor of complement protein that contributes to enhanced evasion of immune functions. It is the most highly polymorphic gene in M1 GAS, a feature that has been exploited in epidemiological and population genetical studies [17-22]. To seek additional evidence supporting the hypothesis that the closely allied M1 isolates from Iceland and Scotland shared a very recent common ancestor, we analysed allelic variation in the *sic* gene using both Illumina assemblies for all 2,009 isolates and hybrid sequenced/assembled closed genomes for the allied isolates. If the phylogenetically allied isolates share a very recent common ancestor, we reasoned that they would have the same unique *sic* allele. In the 2,009 M1 strains, BLASTN search identified *sic* assembled on a single contig for 1,430 strains (71.2%). There were 167 *sic* alleles encoding 167 SIC variants (alleles and variants listed individually in Supplementary Table S2, and given for individual isolates in Supplementary Table S1), consistent with the rapidly evolving hypervariable nature of this gene and protein. Importantly, although 167 alleles were identified, the majority (38 of 50; 76%) of the closely related isolates from Scotland and Iceland had the same *sic* allele (designated *sic0004*), and these were the only isolates to have this allele (Figure 2B). Thus, our *sic* data provided additional molecular evidence that the phylogenetically allied isolates from Scotland and Iceland were related by recent descent.

## Discussion

Although the actual transmission sources and routes are unknown, based on the WGS data currently available, the most parsimonious explanation for the close genetic relationship between the allied M1_UK_ isolates posits direct introduction of a single isolate from Scotland to Iceland, probably by an individual with pharyngitis or an asymptomatic carrier. Iceland is a major tourist destination, with ca 1.9 million foreigners visiting for example in 2022, including 13% from the United Kingdom [23]. Identification of the unexpected genetic link between the isolates from these two countries was made possible by WGS analysis of comprehensive longitudinal samples available from Iceland and Scotland, stressing the importance of reference and public health laboratories in providing new actionable information about isolate dissemination and transmission routes.

The M1_UK_ variant terminology was initially used to describe a lineage that differed from many contemporary circulating M1 strains by 27 shared single nucleotide polymorphisms (SNPs; 26 core and one phage) [1]. This number of SNPs is similar to the magnitude of core genome SNPs differentiating two clades of M1 strains causing human infections in the last decade of the 20th century and first decade of the 21st century [24]. Similarly, an emergent lineage of isolates has been identified in Denmark (n = 97), which differed from all other M1_Global_ and M1_UK_ isolates in Denmark by 15 SNPs in the core genome and was designated M1_DK_ [10]. In the present analysis, we found that all 41 Iceland M1 isolates with the 27 SNPs characteristic of the M1_UK_ lineage differed from all other M1_UK_ (n = 1,293, omitting the nine closely related Scottish isolates) and M1_Global_ (n = 511) isolates by 15 and 43 SNPs in the core genome, respectively. The details of these individual SNPs are appended in Supplementary Table S3. We designated the M1_UK_ sublineage causing infections in Iceland as M1_GLC_ to reflect the historical relationship between the Gaelic populations in Scotland and Iceland.

The genomic data suggest that virtually all M1_GLC_ infections in Iceland were caused by descendants of a single bacterial cell very recently introduced into Iceland from elsewhere, probably Scotland. However, because GAS can be carried asymptomatically and causes large numbers of pharyngitis cases, and because Iceland has a very robust tourism industry, it is not possible to prove the progenitor’s geographical source. The epidemiological data show that the introduction of the M1_GLC_ sublineage into Iceland resulted in rapid dissemination countrywide (data not shown) and an increase in iGAS infections, particularly among young children. We speculate that social distancing practices implemented during the COVID-19 pandemic reduced exposure to GAS, resulting in a reduction in herd immunity that contributed to the 2022 and 2023 surge in iGAS infections. Consistent with this idea, very few cases of iGAS disease occurred in Iceland during the COVID-19 pandemic, from 2020 to early 2022, suggesting that exposure to GAS was less prevalent in Iceland before the surge in iGAS infections between late 2022 and early 2023. Also consistent is the disproportionate number of infections during the surge occurring in young children as they constitute the population with the least prior exposure and developed herd immunity, making them more susceptible to infection on exposure. Given that it is possible to rapidly sequence the genomes of thousands of bacterial isolates for an affordable cost and that Iceland has a small population, strain emergence and dissemination patterns can, with appropriate sampling strategies, be analysed in near real-time, analogous to work done on severe acute respiratory syndrome coronavirus 2 (SARS-CoV-2).

## Conclusion

Our study reveals a public health challenge posed by the emergence and international transmission of *Streptococcus pyogenes* M1 infections between Iceland and Scotland that affected young children disproportionately. Nearly all isolates were of the M1_UK _lineage, which has a high morbidity and mortality, and the derivative M1_GLC_ sub-lineage, underscoring the need for effective surveillance and response strategies. Although our work and others’ demonstrates the public health value of WGS in identifying transmission events, the effectiveness of such efforts is limited, as iGAS infections are not notifiable in all European countries. In addition to the absence of co-ordinated European surveillance programmes capable of real-time data collection and analysis, this limits the capacity for comprehensive assessment and timely public health interventions. Addressing these gaps is crucial for managing iGAS emergence and spread more effectively.

## Supplementary Data

132000 Supplement
